# *Porphyromonas gingivalis-Helicobacter pylori* co-incubation enhances *Porphyromonas gingivalis* virulence and increases migration of infected human oral keratinocytes

**DOI:** 10.1080/20002297.2022.2107691

**Published:** 2022-08-12

**Authors:** Cristopher Soto, Victoria Rojas, Lucas Yáñez, Antonio Hidalgo, Marcela Olivera, Martín Pacheco, Darna Venegas, Daniela Salinas, Denisse Bravo, Andrew F.G. Quest

**Affiliations:** a Cellular Communication Laboratory, Center for Studies on Exercise, Metabolism and Cancer (CEMC), Advanced Center for Chronic Diseases (Accdis), Faculty of Medicine, Universidad de Chile, Santiago, Chile; b Oral Microbiology Laboratory, Department of Pathology and Oral Medicine, Faculty of Dentistry, Universidad de Chile, Santiago, Chile

**Keywords:** *Porphyromonas gingivalis*, *Helicobacter pylori*, hemagglutinins, O-antigen ligase, gingipains, oral keratinocytes migration, toll-like receptor 4

## Abstract

**Background:**

*Porphyromonas gingivalis* is part of the subgingival biofilm and a keystone species in the development of periodontitis. Interactions between *P.gingivalis* and other bacteria in biofilms have been shown to affect bacterial virulence. *Helicobacter pylori* also inhabits the subgingival biofilm, but the consequences of interactions there with *P.gingivalis* remain unknown. Here, we investigated how the pre-incubation of *P.gingivalis* with *H.pylori* affects *P.gingivalis* virulence.

**Methods:**

We assayed *P.gingivalis* internalization by oral keratinocytes (OKs), hemagglutination and biofilm formation to identify alterations in virulence after pre-incubation with *H. pylori*. Also, we evaluated viability and migration of OKs infected with *P. gingivalis,* as well as the role of toll-like receptor 4 (TLR4).   In addition, we quantified the mRNA of genes associated with *P.gingivalis* virulence.

**Results:**

Pre-incubation of *P.gingivalis* with *H.pylori* enhanced *P.gingivalis* biofilm formation, bacterial internalization into OKs and hemagglutination. Infection with pre-incubated *P.gingivalis* increased OK migration in a manner dependent on the O-antigen and linked to  increased expression of the gingipain RgpB. Also, OK TLR4 participates in these events, because upon TLR4 knock-down, pre-incubated *P.gingivalis* no longer stimulated OK migration.

**Discussion:**

We provide here for the first time insight to the consequences of direct interaction between *P.gingivalis* and *H.pylori.* In doing so, we shed light on the mechanism by which *H. pylori* presence in the oral cavity increases the severity or progression of periodontitis.

## Introduction

Periodontitis is the sixth most prevalent inflammatory pathology in humans and constitutes a major health problem worldwide, affecting 30–50% of the adult population [1]. The inflammation causes gradual loss of the structures that support the teeth, including the gingival tissue and alveolar bone, which ultimately results in tooth loss [2]. This inflammatory response is attributable to the presence of dysbiotic periodontal microbiota that accumulate in the subgingival space [3–5].

*Porphyromonas gingivalis*, a Gram negative, coccobacillus, is one of the most studied bacteria associated with periodontitis etiology and progression and has been proposed to represent a key etiological agent of the disease, since it promotes dysbiosis in the subgingival community [6–9]. *P. gingivalis* expresses a wide range of virulence factors, such as adhesion molecules (fimbriae), hemagglutinin A, external membrane molecules like lipopolysaccharide (LPS) or the capsule K antigen, and proteolytic enzymes known as gingipains [10,11].

The subgingival microbiome resides in the periodontal pocket where they form a biofilm. There, physical and metabolic interactions between members of the community including *P. gingivalis*, which coaggregates with other bacteria in the oral biofilm due to the presence of hemagglutinin A and fimbria [12]. Hemagglutinin A specifically, binds to other bacteria, such as *Treponema denticola*, a relevant bacterium in the development of periodontal diseases. Previous studies determined that the coaggregation of both bacteria promotes a symbiotic interaction whereby nutrients are exchanged. Also, an increase in *P. gingivalis* virulence factors involved in adhesion, such as hemagglutinin A and gingipains, is observed as a consequence of this interaction [12–14].

Moreover, the colonization of the oral cavity by bacteria not primarily implicated in periodontitis is now thought to contribute to the dysbiotic processes leading to periodontitis. In this context relevant are observations indicating that *Helicobacter pylori* (*H. pylori*), a microaerophilic Gram-negative bacterium that infects the stomachs of over 50% of the world population and is held responsible for a variety of gastric disorders, also colonizes the subgingival and supragingival space and is present in human saliva [15–17]. The relevance of these findings is underscored by molecular methods and microbiome analysis identifying *H. pylori* as a member of the oral microbiome [18–21]. Furthermore, different studies have suggested that the main extragastric reservoir of *H. pylori* is indeed the oral cavity [22–24], which represents the main route of infection for many pathogens, including *H. pylori*. Bearing this in mind, oral health can be expected to play an important role in determining how *H. pylori* infects and/or re-infects the gastric and oral cavities. Despite this evidence, there are, however, also studies that have failed to confirm the presence of *H. pylori* in the oral cavity [25,26].

*H. pylori* has been shown to interact with *Fusobacterium nucleatum* [27], and recent studies determined that *H. pylori* possesses adhesin proteins that promote hemagglutination via HagA, such as HpaA, SabA and BabA [28,29]. The expression of these proteins is likely to promote the interaction of *H. pylori* with other species of the oral biofilm.

Many clinical studies have correlated the presence of *H. pylori* with periodontitis [22,30–33]. In one study, the presence of *H. pylori* in dental plaques determined that the correlation was better in severe forms of the disease [32]. Also, in another study, periodontal disease was associated with the presence of gastric and oral *H. pylori*. In that report, biopsies from periodontitis patients were found to be positive for *H. pylori* in 70% of the cases. Additionally, 81% of patients with periodontitis were positive for *H. pylori* in oral plaques [33]. Another more recent study identified a correlation between the presence of *H. pylori* in the oral cavity and the progression of periodontitis. There, the presence of *H. pylori* was linked to worse periodontal parameters, such as probing depth, bleeding index and attachment loss [34]. Moreover, several clinical studies have associated poor oral health with the presence of *H. pylori* in the oral cavity [35,36]. Intriguingly, antibiotic treatment against *H. pylori* is linked to a better prognosis in periodontitis patients and vice versa [37,38], reinforcing the notion that *H. pylori* presence in the oral cavity favors progression of this disease.

The presence of *H. pylori* also correlates with greater colonization by periodontitis-associated bacteria in the subgingival pocket and with an increased release of pro-inflammatory cytokines, such as IL-6, IL-8 and TNFα [34]. Presence of the latter is strongly associated with increased apoptosis in OKs, increased permeability of epithelial cells and internalization of periodontal pathogens [39,40]. Moreover, IL-8 promotes the migration of gingival epithelial cells attached to the enamel, promoting thereby the formation or increased depth of periodontal pockets [41]. This cytokine also promotes proliferation and angiogenesis *in vivo* during the inflammatory response triggered during infectious diseases [42]. Different strains of *P. gingivalis* can also promote the secretion of pro-inflammatory cytokines by the activation of TLR4 [43–45], and such activation is not only attributable to *P. gingivalis* LPS but also to other virulence factors, like the fimbriae and gingipains [44,46]. Previous studies from our group found that the infection with *P. gingivalis* increases toll-like receptor 4 (TLR4) mRNA levels, and this increase may relate to some changes in cellular events, like apoptosis [47]. Even though there are no studies that directly link TLR4 activation to the ability to promote migration in OKs, TLR4 activation is known to promote changes in migration in other cells [48–51].

Despite such insight, there are currently no reports on interactions between *P. gingivalis* and *H. pylori* that help us understand how *H. pylori* may promote severity of periodontitis. Thus, the aim of this study was to evaluate the effect of *H. pylori* on the pathogenic potential of *P. gingivalis* (strain W50) following co-incubation, and to determine how these changes modulate the ability of *P. gingivalis* to alter OKs behavior.

## Material and methods

### Bacterial strains and culture conditions

strain W50 (ATCC 53978) was cultured anaerobically at 37°C in enriched brain–heart infusion liquid medium supplemented with hemin 1% (Calbiochem) and menadione 1% (Sigma-Aldrich), or on blood agar plates supplemented with hemin 1% and menadione 1%. The *P. gingivalis* strain PG_1051 corresponds to an isogenic mutant in the PG_1051 gene, which codes for the O-antigen ligase (WaaL), resulting in a strain that lacks the O-antigen region of *P. gingivalis* LPS. This mutant strain was cultured in the same medium supplemented with erythromycin (5 μg/mL). *H. pylori* strain 26695 (ATCC 700392) and the different mutants were cultured in trypticase soy agar plates supplemented with 5% horse serum (Biological Industries), nutritive supplement Vitox (Oxoid) and selective supplement Dent (Oxoid) at 37°C in a humidified atmosphere with 5% CO_2_. All mutant strains for both bacteria are listed in Table 1.

**Table 1.** Bacterial mutant strains used in this study.

Strain	Main characteristic	Reference
Δ*pg1051 (Porphyromonas gingivalis)*	Lacks the OAg region.	[52]
ΔCagA, ΔVacA, ΔGgt *(Helicobacter pylori)*	Lack the CagA, VacA and Gamma-glutamyl transpeptidase proteins, respectively.	[53]
ΔHPU *(Helicobacter pylori)*	Lacks the urease virulence factor.	[54]

The co-incubation liquid medium for *P. gingivalis* W50 and *H. pylori* 26695 was enriched brain–heart infusion liquid medium supplemented with hemin 1%, menadione 1%, 5% horse serum, VITOX and Dent at 37◦C in anaerobiosis. To isolate *P. gingivalis* from the co-cultured medium, 100 μl of the 24 h co-incubation was inoculated into fresh *P. gingivalis* liquid medium (BHI liquid medium supplemented with hemin 1% and menadione 1%, in anaerobiosis, 37°C) and incubated anaerobically for an additional 24 h. After 24 h, an inoculum of the isolation medium was plated on an *H. pylori* agar plate to confirm that no *H. pylori* were present after isolation.

To make growth curves, the optical density of *P. gingivalis* grown in liquid cultures was measured at 560 nm, and solutions were adjusted to contain 1 × 10^4^ bacteria (560 nm = OD 0.7 = 3×10^8^ bacteria). Each dilution was seeded in 1 ml of fresh medium and incubated anaerobically at 37°C for 24 h. Then, an inoculum of 100 μl was taken from each culture, diluted and inoculated on blood agar plates, and colony forming units (CFUs) were counted after 5–7 days of incubation in anaerobiosis. This process was repeated after 24 h for 4 days.

### Cell lines and culture conditions

The immortalized human oral keratinocyte cell line OKF6/TERT2 [52] were incubated in keratinocyte serum-free medium (Gibco) supplemented with bovine pituitary (Gibco), epidermal growth factor (Gibco), calcium chloride solution 0.3 M (Merck) and penicillin/streptomycin (Biological Industries). Cells were incubated at 37°C in 5% CO_2_ atmosphere. *shTLR4* transduced cells were cultivated in the presence of 0,5 μg/ml of puromycin (Gibco). Cells were transduced with lentivirus containing shRNA plasmids against TLR4 (SHCLNG-NM_003266, Sigma-Aldrich) and shScramble (1864, Addgene). Cells were selected for 10 days using 0,5 μg/ml puromycin and *knock down* was confirmed by western blotting.

### Cell infection

OKF6/TERT2 cells were grown as described in each of the next methods. Bacterial cultures were grown anaerobically to an optical density of 0,7 at 560 nm. Then bacteria were washed once with phosphate buffered saline (PBS), suspended in PBS, and added to OKF6/TERT2 cells at a multiplicity of infection (MOI) of approximately 100. After infection for 90 min, cell monolayers were washed and incubated with fresh media supplemented with gentamycin (300 μg/mL) and metronidazole (200 μg/mL) for an additional 24 h (for bacterial internalization, biofilm formation, MTS, trypan blue, western blotting and qPCR assays) or 2 h (for migration assays).

### Biofilm formation assays

*P. gingivalis* liquid cultures were diluted to an optical density of 0.125 at 560 nm, added to a 96-well flat bottom plate in supplemented BHI medium with 1% tryptic soy broth and then incubated anaerobically at 37°C for 48 h. Then, after removing the media by gently inverting the plates, the wells were washed twice with distilled water, avoiding biofilm detachment from the bottom of the wells, and finally plates were left to dry. After 1 h, 100 μL of 0.1% safranin was added to each well and was left for 15 min to stain, followed by two washes in distilled water. Finally, the safranin in the biofilm was eluted with 100 μL of 95% ethanol left for 5 min, and the absorbance of the elution was measured at 490 nm.

### Hemagglutination assays

1 mL of defibrinated horse blood was centrifuged at 3,400 × g for 5 min. The resulting pellet (red blood cells) was washed 3 times and then diluted in PBS to 1% final concentration. In parallel, *P. gingivalis* were grown as described and adjusted to an optical density of 560 nm = 2.0. Then, 200 μL of each suspension were added to one well of a 96-well round-bottom plate. After that, each suspension was serially diluted, by taking 100 μL and mixing with 100 μL PBS (1:2 dilution). This step was repeated until reaching the dilution 1:64. Finally, each well was mixed with an equal volume of 1% sheep erythrocytes and incubated at 37°C for 3 h.

### MTS viability assay

OKT6/TERT2 cells (25,000) were seeded per well in a 96-multiwell plate and incubated for 24 h at 37°C in 5% CO_2_ and infected as described before. Then, the viability of OKF6/TERT2 cells was measured using the MTS reagent ([3-(4,5-dimethylthiazol-2-yl)-5-(3-carboxymethoxyphenyl)-2-(4-sulfophenyl)-2 H-tetrazolium]) in the CellTiter 96 cytotoxicity assay (Promega).

### Trypan blue exclusion assay

OKT6/TERT2 cells (80,000) were seeded per well on a 48-multiwell plate and incubated for 24 h at 37°C in 5% CO_2_ and infected as described before. Then, cells were washed three times with PBS and detached by trypsin–EDTA (Hyclone) incubation for 3 min at 37°C, suspended in Trypan blue solution, and viable cells were counted in a Neubauer chamber using an inverted microscope.

### Bacterial internalization assays

OKT6/TERT2 cells (25,000) were seeded per well on a 96-multiwell plate and incubated for 24 h at 37°C in 5% CO_2_ and infected as described before. Then, the cells were lysed by incubation with 100 μL of saponin (1% w/v) for 10 min. The resulting solution was diluted and inoculated on blood agar plates and colony forming units (CFU) were counted after 5–7 days of incubation in anaerobiosis.

### qPCR

Total RNA was extracted in 1 mL of TRIzol from 2 ml of *P. gingivalis* culture (optical density 560 nm = 1.0) or previously infected OKT6/TERT2 cells (100,000 cells). 0.2 mL of chloroform was added, and then the sample was centrifuged at 15000 × g for 20 min at 4°C. The upper aqueous phase was transferred to a new tube. The RNA was precipitated in 0.5 mL of isopropyl alcohol and 20 g of glycogen at −20°C overnight. Finally, an RNA pellet was obtained by centrifugation at 15000 × g for 20 min at 4°C and was washed twice in 1 ml of 75% ethanol. The pellet was then air-dried and resuspended in RNA/DNase-free water. cDNA was synthesized using a reverse transcription kit following the manufacturer’s recommendations (SuperScrip III, Invitrogen). The mRNA expression of the genes corresponding to the virulence factors to be studied was then measured by qPCR (StepOnePlus; Applied Biosystems). 100 ng of the amplified cDNA was added in each qPCR reaction using SYBR Green (KAPA SYBR Fast qPCR, KAPA Biosystems). The primers used are described in Table 2.

**Table 2.** List of forward and reverse primers used in this study

Gene	Forward primer	Reverse primer
Bacterial 16S	AGGCAGCTTGCCATACTGCG	ACTGTTAGCAACTACCGATGT
rgpB	AGCTCAGGTGCCTACCTTCA	AGCGATCAGCACATCCTTCT
rgpA	CGAATGTCAGATTGCTCGAA	AAGCGTAGGCATCCCTTTTT
kgp	AAGCAAATTCAGGCAGGAGA	GTTGGCACGTACATCGTTTG
PG_1051	TGGCATCGAGCTTCTGTATG	ACAAGGCGACCAAACCATAG
hagA	CCAAAGGTTACGGTTCCTGA	ATCCGAGGGTTTCTTCCAGT
hagC	TTTGCCAAGAATGTGCTGAC	GTCGAGGGCTATGACCTGAG
beta-actin	AAATCGTGCGTGACATTAAGC	CCGATCCACACGGAGTACTT
IL-8	GCTTTCTGATGGAAGAGAGC	GGCACAGTGGAACAAGGACT
TNFα	CAGCCTCTTCTCCTTCCTGAT	GCCAGAGGGCTGATTAGAGA

### Gingipain activity assay

*P. gingivalis* cultures were grown anaerobically to an optical density of 0,7 at 560 nm, then centrifuged at 23,000 × g for 5 min and the supernatant was removed and saved at 37°C. The bacterial pellet was washed with PBS and centrifugated again, repeating this process twice. After that, a solution containing 1 ml of N-a-benzoyl-DL-arginine (BApNA) (0.2 mM) in 50 mM Tris-HCl (pH 7.4), 0.2 mM Dithiothreitol was added to the resuspended bacterial pellets and the supernatants obtained initially. The solution was incubated at 37°C for 30–60 min of reaction, and finally the absorbance of each solution was measured at 405 nm.

### Western blotting

OKs were grown to 70% confluence and infected as described before. Then, cells were rinsed in ice-cold PBS containing 100 μM PMSF, 1 M Na_3_VO_4_ and 1 M NaF. Cells were then centrifuged at 3000 × *g* for 2 min at 4°C and pellets were lysed by sonication in an extraction buffer containing 20 mM HEPES pH 7.4, 0.1% NP-40 and 0.1% SDS, 100 μM PMSF, 1 M Na_3_VO_4_ and 1 M NaF. Protein concentrations in extracts were determined using the BCA protein assay kit. Protein samples were separated by SDS-PAGE (50 μg/lane) for 30 minutes at 90 V and then 90 minutes at 120 V. The gel was transferred to nitrocellulose at 30 V overnight. Then, the membrane was blocked in PBS containing 5% non-fat milk and probed overnight at 4°C with anti-TLR4 (1:5000) (Santa Cruz Biotechnologies) or anti-β-actin (1:5000) (Sigma-Aldrich) antibodies diluted in PBS 5% non-fat milk and 1% Tween-20. After 3 PBS washes, the membrane was probed using goat anti-mouse IgG antibodies coupled to HRP (Bio-Rad) to detect bound first antibodies by EZ-ECL. Protein bands were quantified by densitometric analysis using the ImageJ 1.34 s software.

### Migration assay

OKT6/TERT2 cells (100,000) were seeded in a 24 multi-well plate and incubated for 24 h at 37°C in 5% CO_2_ and infected as described before. For gingipain inhibition assays, the bacterial culture was previously incubated with 50 and 100 μM of Tosyl-L-lysyl-chloromethane hydrochloride (TLCK) for 30 min before the infection. After that, the cells were resuspended in a serum-free medium and added to the top of each Boyden Chamber (Transwell Costar, 6.5 mm diameter, 8 μm pore size), and previously coated with 2 μg*/*ml fibronectin. Also, medium with FBS 5% was added to the bottom chamber to stimulate migration. After 2 h, the inserts were removed, washed gently with PBS, and cells that migrated to the lower side of the inserts were stained with 0.1% crystal violet in 2% ethanol and counted in an inverted microscope. For the migration assays using heat-inactivated *H. pylori*, the inoculum prepared at MOI 100 was previously incubated at 65°C for 2 h and then added to the cell monolayers.

### Statistical analysis

All results were analyzed using the ANOVA test (one-way ANOVA). Dunnett’s multiple-comparison post-tests were used to analyze statistical differences. All groups were from three or more different biological replicates. Differences with p < 0.05 were considered to be statistically significant.

The quantitative reverse transcription-PCR data were analyzed using the MxPro qPCR software (Agilent) and the relative quantification using the 2^−(ΔΔCt)^ method (*P. gingivalis* 16S rRNA was used as prokaryotic control and beta-actin as eukaryotic control). Data were statistically analyzed using PRISM software (version 8.0; GraphPad).

## Results

### *Co-incubation of* P. gingivalis *with* H. pylori

*H. pylori* was previously shown to be present in the subgingival biofilm where it can interact with members of the oral cavity, such as *Fusobacterium nucleatum* [27]. Moreover, *H. pylori* presence is associated with increased relative abundance of bacteria, such as *P. gingivalis* [34]. Thus, in order to determine whether these effects may be attributable to the ability of *H. pylori* to interact with *P. gingivalis*, we first sought to generate culture conditions permitting the growth of both *H. pylori* and *P. gingivalis*. We found that an enriched brain–heart infusion liquid medium supplemented with hemin 1%, menadione 1%, horse serum 5%, VITOX and Dent at 37◦C in anaerobiosis permitted the growth and viability of both bacteria up to 24 h, using a 1:1 initial inoculum (Figure S1A). We also found that *H. pylori* did not grow under *P. gingivalis* culture conditions (enriched brain–heart infusion medium supplemented with hemin 1% and menadione 1% in anaerobiosis), so we used *P. gingivalis* culture conditions to select for and recover exclusively *P. gingivalis* from the co-incubation (Figure S1B). After 24 h of co-incubation, *P. gingivalis* was isolated, and the corresponding assays were performed.

### *Virulence of* P. gingivalis *co-incubated with* H. pylori

There is evidence that bacteria like *T. denticola* can interact with *P. gingivalis* and increase *P. gingivalis* virulence [14], but no studies are available analyzing how interaction between *P. gingivalis* and *H. pylori* may increase *P. gingivalis* virulence and periodontitis severity. Hence, to evaluate the effect of *H. pylori* on *P. gingivalis* virulence after co-incubating both bacteria, we performed biofilm formation, hemagglutination and cell internalization assays.

**Figure 1.**
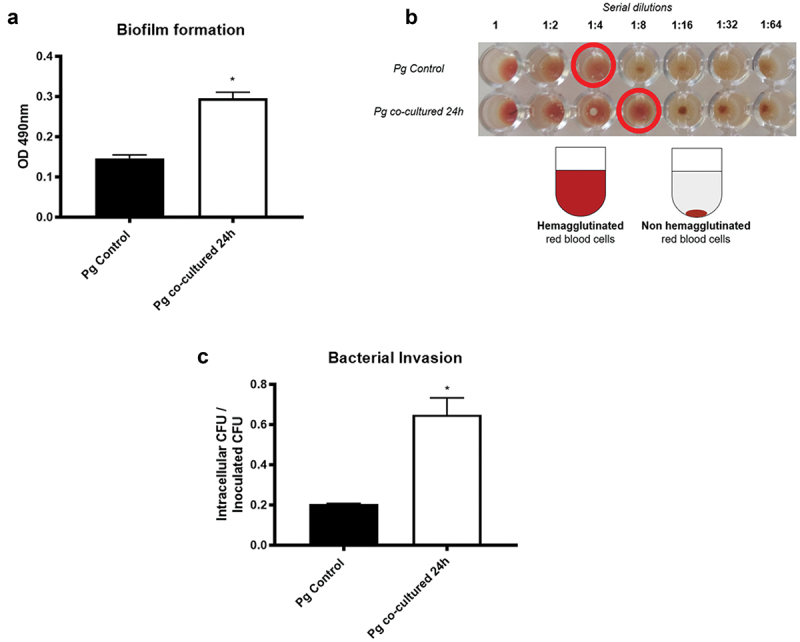
**Co-incubation with *H. pylori* increases *P. gingivalis* virulence *in vitro.*** (A) Optical density at 490 nm of the dye (safranine) retained by the biofilms formed (for 48 h) by *P. gingivalis* without co-incubating and by *P. gingivalis* co-incubated with *H. pylori* for 24 h is shown (n = 3). (B) A representative figure (n = 3) of the hemagglutination of horse red blood cells by *P. gingivalis* controls and those co-incubated with *H. pylori* for 24 h. The values observed in the upper part correspond to the ratio of dilutions made from bacterial cultures with an optical density of 1.5 at 560 nm (corresponding to the initial value of 1). Red circles indicate the last hemagglutinated well in each condition. On the right, a schematic figure showing a hemagglutinated and a non-hemagglutinated well. (C) Invasion of OKF6/TERT2, expressed as the ratio (intracellular CFU/inoculated CFU) of *P. gingivalis* controls to those co-incubated with *H. pylori* for 24 h. One-way ANOVA, Dunnett post test. * indicates significant differences of p < 0.05. n = 3.

Biofilm formation assays were evaluated by measuring the optical density of the safranin stain retained by the biofilm after 48 h of bacterial growth. *P. gingivalis* previously co-incubated with *H. pylori* for 24 h increased 2.1-fold the amount of biofilm compared to the *P. gingivalis* monoculture (Figure 1A).

Hemagglutination assays were evaluated by preparing serial dilutions of the bacteria and mixing with them equine red blood cells to observe a precipitation of the red blood cells when they were not hemagglutinated, or the formation of a suspended, gelatin-like substance when they were hemagglutinated. We observed that co-incubated *P. gingivalis* induced hemagglutination of the red blood cells up to a dilution of 1:8, while monocultured *P. gingivalis* only did so up to the 1:4 dilution. This result indicates that less co-incubated *P. gingivalis* were necessary to promote hemagglutination of red blood cells compared to the monocultured *P. gingivalis* (Figure 1B).

To compare the ability of *P. gingivalis* grown in monoculture or co-incubated to infect OKs, cellular internalization assays were performed. Our results show that *P. gingivalis* co-incubated with *H. pylori* increased 3.2-fold internalization by OKs compared to the *P. gingivalis* in monoculture (Figure 1C). Together, these results indicate that *P. gingivalis* co-incubation with *H. pylori* increased *P. gingivalis* virulence.

Finally, and to determine the virulence factor in *H. pylori* responsible for the changes generated in *P. gingivalis*, we evaluated the effects of four classical *H. pylori* virulence factors (VacA, CagA, Gamma Glutamyl Transpeptidase (GGT) and Urease (HPU)) using mutant strains in co-incubation experiments with *P. gingivalis*. Subsequently, *P. gingivalis* was isolated from the co-incubation and biofilm formation tests were performed. The results showed that for none of these *H. pylori* mutant strains the change in *P. gingivalis* behavior was different from that observed following co-incubation with the wild type *H. pylori* strain (Figure S2).

### P. gingivalis *co-incubated with* H. pylori *increases mRNA levels of* P. gingivalis *virulence factors.*

To characterize the changes in virulence observed following the co-incubations with *P. gingivalis*, we analyzed the mRNA levels of *hag* genes, which are important for bacteria–bacteria interaction, biofilm formation, adhesion and internalization by oral keratinocytes [53,54]. Also, we evaluated the mRNA levels of gingipains (*kgP, rgpA* and *rgpB* genes), important for the secretion of pro-inflammatory cytokines and cell migration [55,56]. In addition, we evaluated the expression of the O-antigen ligase gene to identify changes associated with apoptotic processes [47].

**Figure 2.**
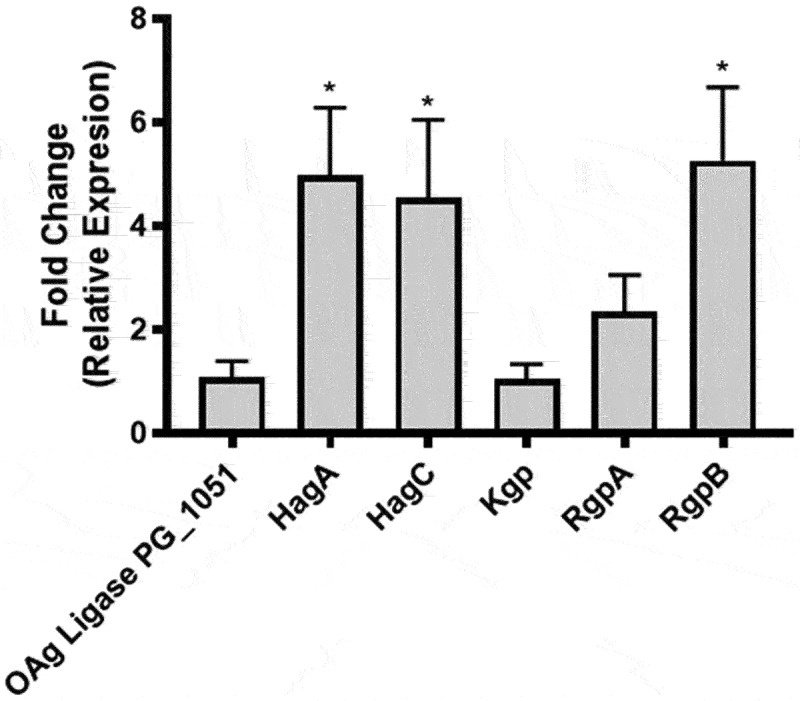
**The co-incubation with *H. pylori* increases mRNA levels of some *P. gingivalis* virulence genes**. The fold change of the relative expression (∆∆Ct) of the virulence genes of *P. gingivalis* are shown. The expression of co-incubated *P. gingivalis* and *P. gingivalis* controls is compared. A specific region in 16S rRNA of *P. gingivalis* was used as the reference gene. One-way ANOVA, Dunnett post-test. * indicates significant differences of p < 0.05. n = 3.

Our results show that the mRNA levels of hemagglutinins HagA and HagC, as well as the arginine gingipain RgpB of *P. gingivalis* increased (5-, 4.6- and 5.3-fold, respectively) following co-incubation, as compared with the levels observed for *P. gingivalis* grown in monoculture (Figure 2). There were no significant changes in the relative expression of *PG_1051, kgP and rgpA*. These results indicate that *P. gingivalis* co-incubation with *H. pylori* increases of the expression of *hagA, hagC* and *rgpB* genes in *P. gingivalis*.

### P. gingivalis *co-incubated with* H. pylori *increases mRNA and proteins levels of pro-inflammatory cytokines in OKs.*

**Figure 3.**
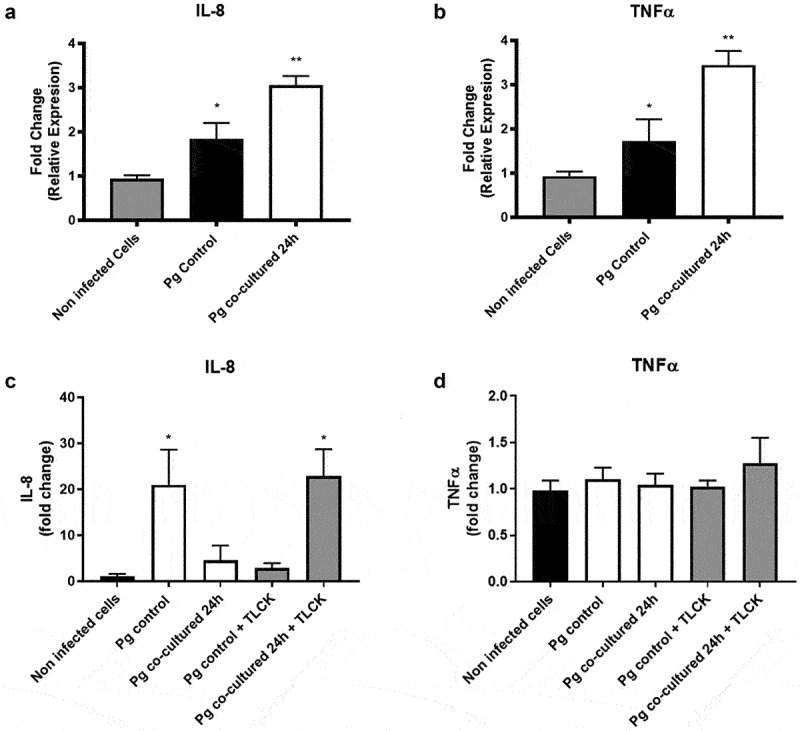
***P. gingivalis* co-incubation with *H. pylori* increases the expression of IL-8 and TNFα mRNA levels, and IL-8 protein level**. The fold change of the relative expression (∆∆Ct) of IL-8 (A) and TNFα (B) genes from cells infected with *P. gingivalis* are shown. The expression in OKF6/TERT2 cells infected with co-incubated *P. gingivalis* and control *P. gingivalis* is compared with uninfected cells. Beta-actin was used as the reference gene. The fold change of IL-8 (C) and TNFα (D) concentration in the supernatant of OKF6/TERT2 cells infected with co-incubated *P. gingivalis* or control *P. gingivalis* is shown. The fold change is compared with respect to uninfected cells = 1. One-way ANOVA, post-test Dunnett. * indicates significant differences of p < 0.05 and ** of p < 0.001. n = 3.

*P. gingivalis* can promote the expression and secretion of different interleukins, like IL-8 and TNFα (Brat et al., 2005; Fotin-Mleczek et al., 2004; Wajant et al., 2003), which also contribute to the progression of periodontitis [39–41]. To evaluate if co-incubating *P. gingivalis* with *H. pylori* changed the expression of IL-8 and TNFα, we analyzed mRNA levels of both cytokines in OKs infected with either monocultured or co-incubated *P. gingivalis*. As expected, based on the literature, infection with the *P. gingivalis* monoculture increased the expression of both cytokines with respect to the non-infected cells (1.8 and 1.7-fold, respectively); however, this increase was significantly greater when OKs were infected with co-incubated *P. gingivalis* (3.1- and 3.4-fold, respectively) (Figure 3A and 3B). To evaluate whether the increment in mRNA led to an increase in the protein concentration of IL-8 and TNF, ELISA assays were performed. Our results revealed that no changes in TNFa protein levels were detected as compared to the uninfected controls when analyzing cells infected with monocultured or cocultured *P. gingivalis* (Figure 3D). For IL-8, on the other hand, we observed an increase in the expression when infected with monocultured bacteria compared to uninfected cells. Surprisingly, however, such an increase was not observed when infecting with cocultured bacteria (Figure 3C). Since gingipains have been shown to degrade cytokines both in the extracellular medium and in the cytoplasm [56], we repeated the assay in the presence of a gingipain inhibitor (TLCK) and observed that in the presence of TLCK IL-8 became readily detectable in the culture medium (Figure 3C).

In summary, therefore, *P. gingivalis* co-incubation with *H. pylori* promoted *P. gingivalis* virulence. Moreover, upon infection of OKs by such co-incubated *P. gingivalis*, the mRNA levels of IL-8 and TNFα increased significantly more and for IL-8 this translated into increased protein levels when gingipains were inhibited.

### P. gingivalis *co-incubated with* H. pylori *increases migration of infected OKs.*

**Figure 4.**
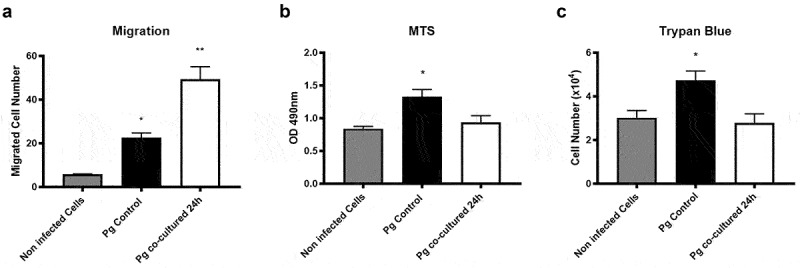
**Changes in viability and migration of oral keratinocytes infected with *P. gingivalis* mono-cultures or co-incubations with *H. pylori.*** (A) Migration of OKF6/TERT2 after 2 h are shown, following infection for 2 h with *P. gingivalis* controls or *P. gingivalis* co-incubated with *H. pylori* 24 h. Data correspond to the average number of migrated cells observed in 7 fields. (B) Optical density at 490 nm to determine viability of OKF6/TERT2, after infection with *P. gingivalis* controls and *P. gingivalis* co-incubated with *H. pylori* for 24 h, by MTS assays. Results shown were obtained 24 h post-infection. (C) Number of viable OKF6/TERT2 (Trypan Blue assay), after being infected with *P. gingivalis* controls and *P. gingivalis* co-incubated with *H. pylori* for 24 h. Results shown were obtained 24 h post-infection. ONE-way ANOVA, Dunnett post-test. * indicates significant differences of p < 0.05 and ** of p < 0.001. n = 3.

It has been reported that IL-8 and TNFα enhance migration of different cell lines [41,57,58]. Since we observed a greater increase in the mRNA of both cytokines in cells infected by co-incubated *P. gingivalis*, we evaluated cell migration, proliferation, and viability of infected cells. As expected, *P. gingivalis* monoculture increased the migration of infected OKs (3.8-fold), but this increase was higher when cells were infected with co-incubated *P. gingivalis* (8.2-fold) (Figure 4A).

As we previously observed that *P. gingivalis* increases the viability and proliferation of infected OKs [47], we evaluated cell viability and proliferation of OKs infected by *P. gingivalis* co-incubated with *H. pylori*. We observed that the *P. gingivalis* monoculture increased cell viability and proliferation, as previously reported by our group [47]. However, *P. gingivalis* co-incubated with *H. pylori* did not promote any change in these characteristics (Figures 4B and 4C).

These results confirm that monocultured *P. gingivalis* increase the migration of OKs, but also that *P. gingivalis* co-incubated with *H. pylori* are even more effective at promoting the migration of infected OKs.

### *Gingipains from* P. gingivalis *promote the increase in migration of infected OKs.*

**Figure 5.**
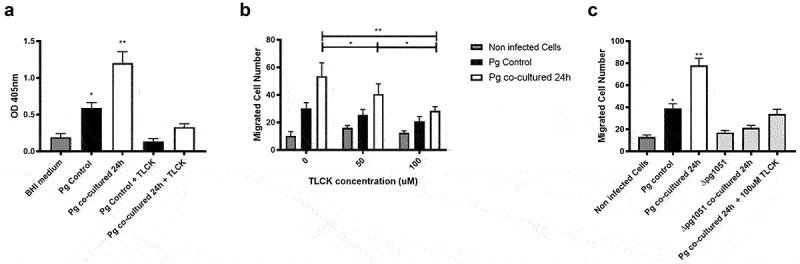
**Gingipains from *P. gingivalis* increase the migration of infected oral keratinocytes**. (A) Optical density at 405 nm to measure gingipain activity. TLCK 100 uM was used as a gingipain inhibitor. (B) Migration of OKF6/TERT2 cells after 2 h are shown, following infection for 2 h with *P. gingivalis* control and *P. gingivalis* co-incubated with *H. pylori* 24 h (both previously incubated with inhibitor of gingipains, TLCK). Data correspond to the average number of migrated cells observed in 7 fields. (C) Migration of OKF6/TERT2 cells after 2 h are shown, following infection for 2 h with *P. gingivalis* controls or co-incubated with *H. pylori* for 24 h. Additionally, the effects of ∆pg1051, a mutant *P. gingivalis* strain lacking the o-antigen ligase enzyme, alone or after co-incubation with *H. pylori* for 24 h, are shown. Data correspond to the average number of migrated cells observed in 7 fields. One-way ANOVA, Dunnett post-test compared against BHI medium and non-infected cells (A and C, respectively); Two- way ANOVA, Bonferroni post-test (B) * indicates significant differences of p < 0.05 and ** of p < 0.001. n = 4.

It was reported that both gingipains and cytokines, like IL-8 and TNFα, can promote cell migration [55]. Since our results revealed an increase in RgpB mRNA and cell migration, we decided to evaluate the role of gingipains in the migration of OKs infected by *P. gingivalis* co-incubated with *H. pylori*, in the presence of a chemical gingipain inhibitor (TLCK). We first confirmed that TLCK was able to inhibit gingipains, using N-a-benzoyl-DL-arginine (BApNA), a substrate for gingipains that generates a colored compound upon proteolytic processing. We observed that co-incubated *P. gingivalis* promote a greater increase in the gingipain activity compared with mono-cultured *P. gingivalis* (Figure 5A). Moreover, the migration assays showed that TLCK at concentrations of 50 mM and 100 mM reduced OK migration promoted by co-incubated *P. gingivalis*, (Figure 5B).

Intriguingly, it was reported that the O-antigen region of the *P. gingivalis* LPS is required for the activation of arginine gingipains and could be relevant in the processing of the gingipains to permit their final activation [59,60]. Thus, we used a *P. gingivalis* strain lacking the O-antigen region (*Δpg1051*), as the consequence of introducing an isogenic mutation in the gene coding the O- antigen ligase (*PG_1051*). As expected, for the mutant strain *Δpg1051* co-incubation with *H. pylori* did not increase the migration of infected OKs beyond values observed for the non-infected cells or those monocultured with the *Δpg1051 *mutant (Figure 5C).

Together, these results strongly suggest that gingipains enhance the migration of oral keratinocytes infected by *P. gingivalis* co-incubated with *H. pylori* and that their activity is key to promoting these changes.

### The increase in migration of infected OKs is mediated by TLR4

**Figure 6.**
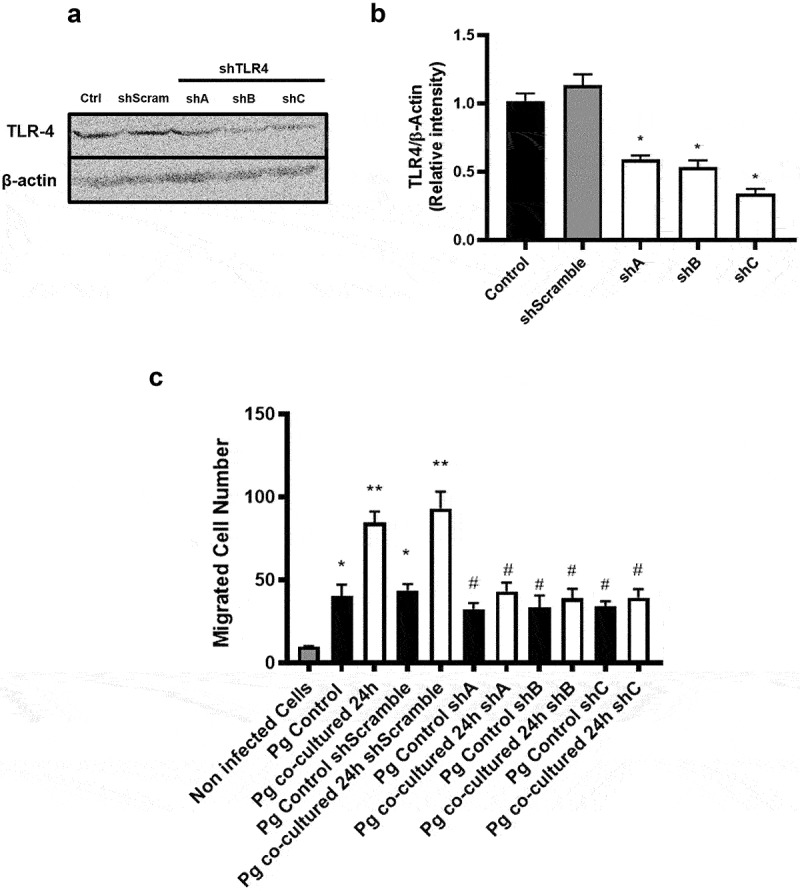
**TLR4 is required to increase the migration of infected oral keratinocytes**. (A) Representative blot showing TLR4 and β-actin protein levels in control OKF6/TERT2 cells and cells transduced with shRNA against TLR4. (B) Quantification by scanning densitometry of the data obtained by western blotting and normalization of TLR4 to b-actin levels. (C) Migration of OKF6/TERT2 control cells and OKF6/TERT2-shTLR4 cells after 2 h are shown, either following infection for 2 h with *P. gingivalis* controls or *P. gingivalis* co-incubated with *H. pylori* 24 h. Data correspond to the average number of migrated cells observed in 7 fields. One-way ANOVA, Dunnett post-test * indicates significant differences of p < 0.05, ** of p < 0.001 (against non-infected cells) and # of p < 0,05 (against *P. gingivalis* co-incubated 24 h). n = 3.

Given that TLR4 activation induces pro-inflammatory cytokine release [43–45] and promotes migration in gastric epithelial cells, glioma cells, prostate cancer cells and vascular smooth muscle cells [48–51], we evaluated the behavior of OKs transduced with a plasmid containing a shRNA against TLR4 in migration assays after infection with *P. gingivalis*. Our results revealed that the transduction with TLR4-specific shRNA decreased TLR4 protein levels by roughly 60% (Figure 6A and 6B). Moreover, while co-incubated *P. gingivalis* increased the migration of OKs in comparison with non-infected cells, this increase did not occur when infecting TLR4 knock-down OKs with co-incubated *P. gingivalis* (Figure 6C).

## Discussion

The oral cavity is home to many bacteria. Several studies estimate that more than 700 bacterial species reside in this cavity and that many of them constitute part of a structured subgingival biofilm in which individual species may interact due to the presence of adhesins and receptor proteins that promote bacteria–bacteria interactions [6,61].

Both *P. gingivalis* [6–9] and *H. pylori* [16,17,62] reside in the subgingival biofilm, and both possess membrane proteins that allow them to interact with and bind to other bacteria in the same community [12–14,27–29]. Therefore, it was to be suspected that interactions between both bacteria may occur, especially after the studies showing not only that *H. pylori* is present in saliva and dental plaques [18–20], but also is part of the oral microbiome and subgingival biofilm, where interactions via adhesins with other members of the community, such as *F. nucleatum*, have been observed [63]. Furthermore, studies have linked the presence of *H. pylori* to poorer periodontal health and an increase in the relative abundance of other bacteria, such as *P. gingivalis* [34]. Andersen et al. even argued that due to the large amount of adhesins that *H. pylori* possesses on its surface, such as HpaA, SabA and BabA, and normally uses for anchorage and adhesion to the epithelial cells of the stomach cavity, it may not come as a surprise that these same proteins could promote *H. pylori* colonization of the subgingival biofilm. However, prior to this study, there was no direct evidence available connecting both bacteria by showing that co-incubation *in vitro* altered the virulence of *P. gingivalis*.

In the present study, we developed a co-incubation medium containing BHI, hemin, menadione, VITOX and equine serum that permitted the growth of *P. gingivalis* and *H. pylori* up to 48 h. It would be interesting to determine if an extended co-incubation time could promote even greater changes in the virulence of *P. gingivalis*, but the controls allow us to suggest that the main reason why viable *H. pylori* were not recovered after 48 h is due to the strict anaerobiosis co-incubation conditions that need to be employed to recover viable *P. gingivalis*. It is important to mention that there were no changes in the proliferation rate of *P. gingivalis* in co-incubation, given that it maintains growth curves without significant changes compared to the monoculture. Additionally, we could rule out that viable *H. pylori* were present in the medium used to infect the cells, because *H. pylori* did not grow in the medium used for *P. gingivalis* (Figure S1B), which allowed us to easily isolate *P. gingivalis* from the co-incubation.

The co-incubation conditions revealed that the cultivation of both bacteria together lead to important changes in the virulence of *P. gingivalis* as evidenced by processes like biofilm formation, hemagglutination and internalization capacity. This raised the question whether there were any changes in the expression of proteins related to *P. gingivalis* virulence, such as hemagglutinin A, which allows *P. gingivalis* to recognize, adhere to and internalize into cells. *P. gingivalis* gingipains have been strongly associated with the release of cytokines from epithelial cells and migratory processes and are important in the generation of the periodontal pocket in the early stages of periodontitis [41,55,64]. LPS, specifically the O-antigen ligase of *P. gingivalis*, is associated with modulation of apoptosis in infected epithelial cells [47].

It should be noted that the results obtained in the biofilm tests were somewhat surprising, because they indicate that the bacterial biomass, as reflected in augmented safranin retention, actually increases under these conditions. However, the growth curves for the liquid co-cultures of *P. gingivalis* did not reveal significant changes in proliferation. Thus, it is intriguing to speculate that a mechanism(s) associated with biofilm formation might exist permitting the bacteria to selectively grow better when present in such structures. If indeed the case, the possible underlying mechanisms remain to be determined. Also, due to the period of time that exists between the co-culturing of *P. gingivalis* the different experiments that follow using *P. gingivalis* isolated from the co-culture, one must assume that a mechanism(s) exists that allows the effects of co-culturing to persist over time. Moreover, one may speculate that changes in gene expression occur during co-culturing and probably explain this phenomenon. With this in mind, we are currently carrying out transcriptomics/proteomics experiments to characterize these changes in *P. gingivalis*.

qPCR results identified an increase in the expression of genes, such as *hagA, hagC* and *rgpB*. Particularly, the increased expression of *hagA* and *hagC* likely relate to the increase in bacterial invasion, biofilm formation and hemagglutination seen in *in vitro* assays, because hemagglutinin presence is strongly linked to bacterial adherence and intracellular invasion [53,54]. In addition, the detected increase in *rgpB* mRNA correlates with an increase in the proteolytic activity of the enzyme, which may contribute to changes in cytokine degradation [65,66] and increased migration [55,56].

We observed that infection with *P. gingivalis* increased OK migration even without former exposure to *H. pylori*, but that this increase was significantly greater in cells infected with the co-incubated *P. gingivalis*. Such increased OK migration can be associated with different clinical symptoms linked specifically to the progression and severity of periodontal disease, including an increase in the depth of the periodontal pocket or regression of gingival tissue. In contrast, *H. pylori* alone did not alter migration, which allows us to exclude the possibility that the results obtained may have been affected by the presence of *H. pylori*. Also, we noted that viable *H. pylori* are required in the co-incubation process to promote changes in migration induced by *P. gingivalis* infection of cells (Figure S3). Furthermore, we determined that co-incubating increased the expression of IL-8 and TNFα mRNA. Together with the increase in the expression of *rgpB*, this may explain the higher rate of migration, as suggested by previous studies that correlate both cytokine and gingipain expression with changes in the migration of OKs and other cell lines [55,67]. We also determined that TLR4 participates in promoting the migration of infected OKs, so understanding the signaling pathway(s) activated by this receptor, should yield insight into these processes and potentially also to improving current treatments.

Until now, the precise manner by which *H. pylori* might induce changes in *P. gingivalis* remained unknown. However, co-incubation with *H. pylori* mutant strains lacking CagA, VacA, urease (HPU) or γ-glutamyl transferase (GGT), the main virulence factors of *H. pylori*, had the same effects as coincubation with wildtype 26695 strain, suggesting that these *H. pylori* virulence factors are not relevant in this context.

In summary, in this study we developed a co-incubation system to show that co-habitation of *H. pylori* with *P. gingivalis* increases the virulence of *P. gingivalis* and the expression of several virulence genes. These changes promote not only an increase in the expression of pro-inflammatory cytokines, such as IL-8 and TNFα, but also an increase in the migration of infected OKs, which our observations attribute to the enhanced expression of *P. gingivalis* RgpB gingipain. These results are important because we used a model in which we evaluated the effects of bacterial interactions, similar to those that may occur in the subgingival biofilm, where hundreds of different species coexist. Furthermore, we provide for the first time insight into the consequences of the direct interaction between *P. gingivalis* and *H. pylori*. Finally, we shed light on the mechanism by which *H. pylori* increases the severity or progression of periodontitis upon colonization of the oral cavity. Given the prevalence of *H. pylori* worldwide in the human population, this is certainly an aspect that merits further consideration when contemplating public health policies.

## Supplementary Material

Supplemental Material
