# Supplementary material for: Porphyromonas gingivalis-Helicobacter pylori co-incubation enhances Porphyromonas gingivalis virulence and increases migration of infected human oral keratinocytes
Source: J Oral Microbiol. 2022 Aug 12;14(1):2107691. doi: 10.1080/20002297.2022.2107691 (PMC9377229; doi:10.1080/20002297.2022.2107691)
Supplement: Supplemental Material [file ZJOM_A_2107691_SM8283.docx]

Supplementary Material

## Supplementary Figures

**A**

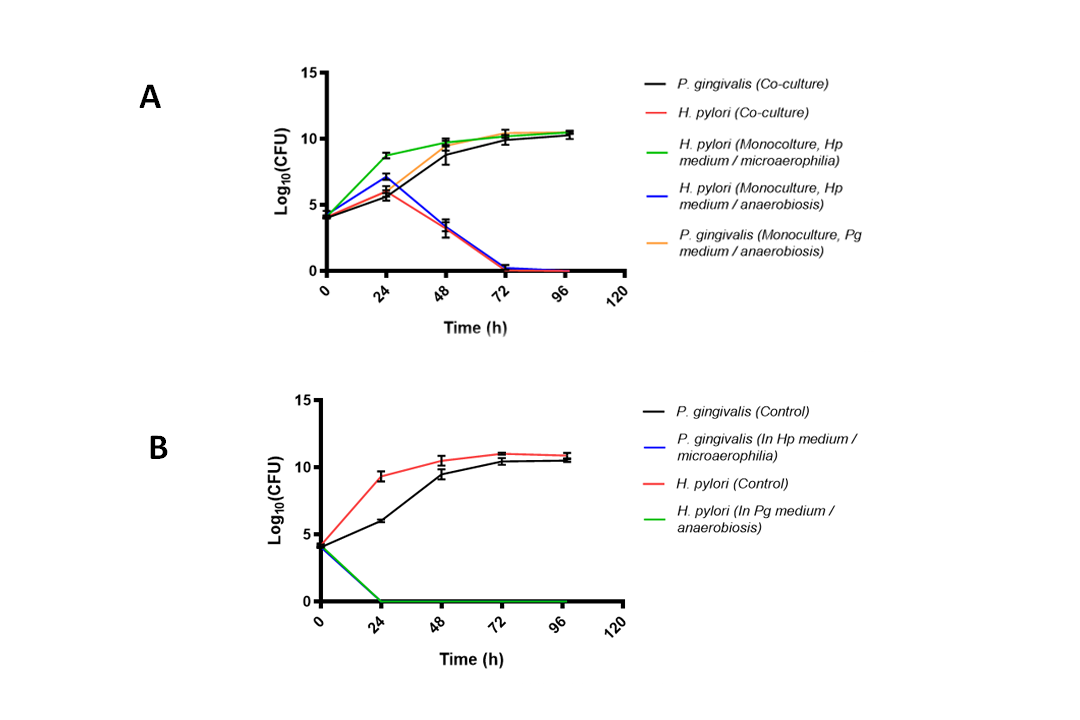


**B**

**Supplementary Figure 1: Co-culture and monocolture growth curves for *H. pylori* and *P. gingivalis.*** (A) Growth curves of *H.pylori 26695* and *P. gingivalis* W50 grown individually or in co-culture. *H. pylori* mono-cultures grown in microaerophilia or anaerobiosis are shown as controls. (B) Growth curves of *P. gingivalis* and *H. pylori* under normal conditions (control) and when grown under conditions used for the other bacteria.


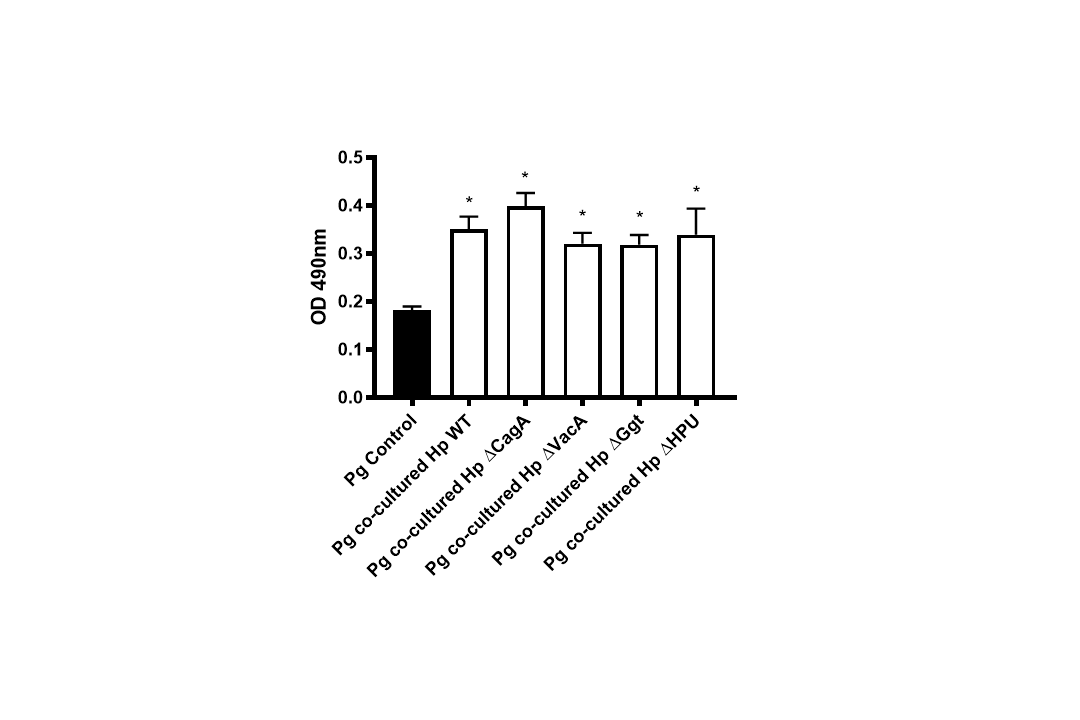


**Supplementary Figure 2: *P. gingivalis* co-cultured with wild-type or mutant *H. pylori*, increases the ability to form a biofilm**. The optical density at 490 nm of the dye (safranin) retained by the biofilms formed (for 48 h) by *P. gingivalis* control and by *P. gingivalis* co-cultured with wild type *H. pylori* and with *H. pylori* mutants for 24 h is shown. One-way ANOVA, Dunnett post-test. * indicates significant differences of p <0.05. n = 3


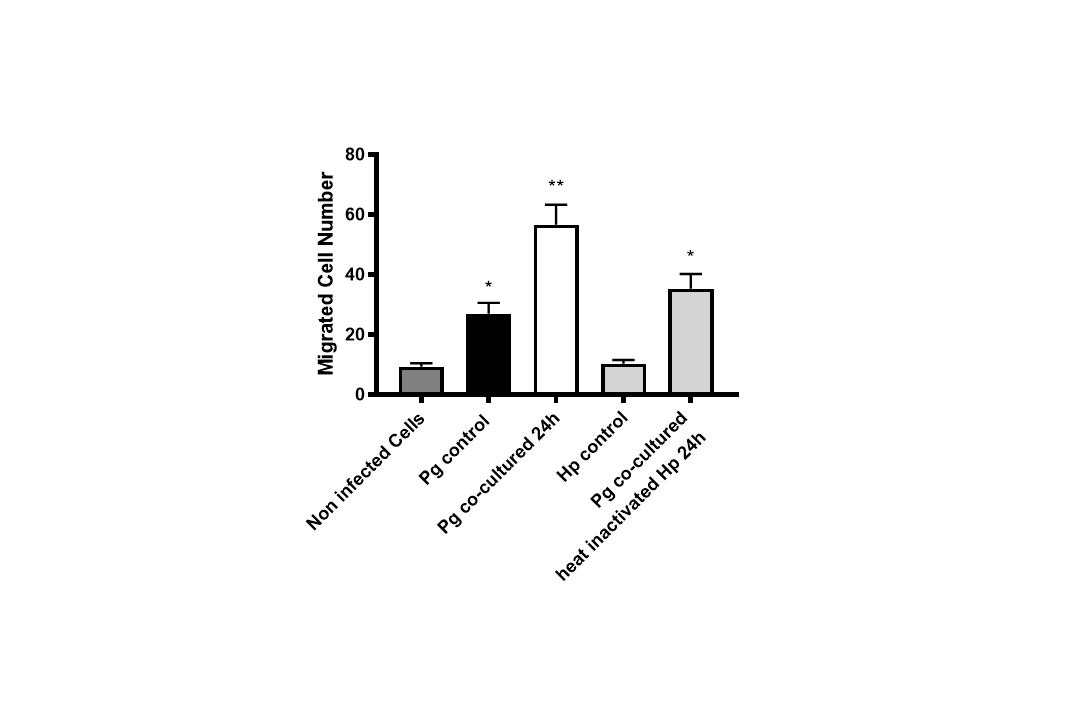


**Supplementary Figure 3: Migration of gingival epitelial cells infected with *H. pylori* and *P. gingivalis* co-cultured with heat inactivated *H. pylori.*** Migration of GECs after 2 h is shown, either following infection for 2 h with *P. gingivalis* control, or *P. gingivalis* co-cultured with *H. pylori* 24 h or *H. pylori* monoculture control or *P. gingivalis* co-cultured with heat inactivated *H. pylori* 24 h. Data shown are equivalent to the average number of migrated cells observed in 7 fields. ONE-way ANOVA, Dunett post-test. * indicates significant differences of p <0.05 and ** of p<0.001. n = 3
